# Psychophysiologic symptom relief therapy for chronic back pain: hypothesis and trial rationale

**DOI:** 10.3389/fpain.2024.1328495

**Published:** 2024-07-18

**Authors:** Myrella Paschali, Garrett S. Thompson, Shivani Mehta, Patricia M. Howard, Jolin B. Yamin, Robert R. Edwards, Michael W. Donnino

**Affiliations:** ^1^ Department of Anesthesiology, Harvard Medical School, Brigham & Women’s Hospital, Chestnut Hill, MA, United States; ^2^ Department of Emergency Medicine, Beth Israel Deaconess Medical Center, Boston, MA, United States; ^3^ Division of Pulmonary, Critical Care and Sleep Medicine, Beth Israel Deaconess Medical Center, Boston, MA, United States

**Keywords:** chronic pain, mind-body therapy, psychophysiology, back pain, back

## Abstract

Chronic pain syndromes affect over one-third of the US adult population and often lead to significant disability and a reduced quality of life. Despite their high prevalence, causal links between chronic pain syndromes and anatomic abnormalities are often not apparent. Most current chronic pain treatments provide modest, if any, relief. Thus, there is a pressing need to understand the causal mechanisms implicated in chronic pain as a means to develop more targeted interventions for improvement in clinical outcomes and reduction in morbidity and financial burden. In the present manuscript, we summarize the current literature on treatment for chronic pain, and hypothesize that non-specific chronic back pain (without a clear organic etiology, such as tumors, infections or fractures) is of psychophysiologic origin. Based on this hypothesis, we developed Psychophysiologic Symptom Relief Therapy (PSRT), a novel pain reduction intervention for understanding and treating chronic pain. In this manuscript, we provide the rationale for PSRT, which we have tested in a pilot trial with a subsequent larger randomized trial underway. In the proposed trial, we will evaluate whether non-specific chronic back pain can be treated by addressing the underlying stressors and psychological underpinnings without specific physical interventions.

## 1 Background

Chronic pain syndromes affect nearly 100 million adults in the United States and can substantially impair quality of life (1). Chronic pain is strongly associated with depression, anxiety, fatigue (2–4) and a lower socioeconomic status (5). In addition, chronic pain has major economic consequences, with the estimated annual cost to be approximately $560–635 billion, in the United States alone, including health care expenses and lost productivity (1).

Current models of chronic pain syndromes do not sufficiently explain the etiology for many disorders including back pain. Without an identifiable organic etiology, these pain syndromes are deemed “non-specific”. While components of chronic low back pain have been associated with anatomic abnormalities (e.g., degeneration of intervertebral discs), a definitive causal link has not been proven for many of these abnormalities (6). Previous reports from as early as 1946 question the role of focal anatomic or physiologic abnormalities in leading to and sustaining a state of chronic pain (7–9). Another proposed mechanism related to chronic pain are trigger points. Trigger points are prevalent in fibromyalgia and myofascial pain syndrome and are believed to emerge from both physical factors (e.g., muscle overuse or trauma and abnormal structural factors) and psychological factors (e.g., stress) (10). In this model, there is a bidirectional relationship between physical and psychological factors (11). Current methodology in both pain management and research is to seek physical origins of pain alone or in combination with adjunctive psychological therapy. In the current manuscript, we propose a paradigm shift in the search for and treatment of the underlying etiology of non-specific chronic back pain. Our overall scientific premise is that chronic back pain without clear organic etiology, such as tumors, infections, ankylosing spondylitis, or fractures, are of psychosomatic or psychophysiological nature. Therefore, psychological processes precipitate and perpetuate the development of the pain. We further hypothesize that this condition can be treated by addressing underlying stressors, psychological underpinnings, and fear-avoidance behaviors that perpetuate chronic pain. This concept has previously been proposed in the lay press by Dr. John Sarno but has not been tested rigorously in well-controlled clinical trials (12). Hence, we developed a psychological intervention, Psychophysiologic Symptom Relief Therapy (PSRT), based in large part on Dr. John Sarno's work, to target psychophysiologic sources of chronic pain. Furthermore, we provide a detailed rationale for PSRT as tested in our pilot study and now being evaluated in a larger, randomized clinical trial.

## 2 The physical paradigm of pain, current etiological models, and treatment options for chronic pain

### 2.1 The traditional paradigm: pain is caused by a physical lesion

The traditional paradigm for describing the etiology of pain is grounded in the hypothesis that physical injury leads to an excitement of peripheral nerve fibers which transmit a signal to the central nervous system leading to the subsequent feeling of pain. Thus, the amount of pain is expected to be associated with and proportional to the nature and degree of tissue damage. While this makes intuitive sense and is often the case for acute pain (burns, fractures, cuts, severe sprains etc.) and some cases of chronic pain (cancer, chronic infection, etc.), this concept may not broadly apply to all chronic pain. The clinical workup of chronic low back pain often includes a number of diagnostic tests including computed tomography (CT) and/or magnetic resonance imaging (MRI). Imaging often either identifies multiple non-specific findings or identifies minor findings with no pathological significance that subsequently do not match or explain the patients' symptoms (6). Jensen et al. conducted MRI examinations on individuals without back pain, finding that 52% of participants in this cohort had disk bulges and/or protrusions on imaging (8). In a meta-analysis published in *Spine*, Endean et al. evaluated 45 published studies assessing disc protrusion, nerve root displacement/compression, disc degeneration, and high intensity zones concluding that individually, none of these abnormalities provide a clear indication that LBP (low back pain) is attributable to underlying pathology” (6). Other studies have found no relationship between the degree of abnormal MRI findings and the degree of disability or intensity of low back pain (13).

### 2.2 Current etiological models of chronic pain

In the past, psychological factors were not routinely assessed and were considered to be irrelevant to pure physiological pain. However, over the past 4 decades, research has consistently shown that pain is caused by a complex biopsychosocial phenomenon (14). This biopsychosocial model describes pain as a multifaceted interaction between physiological, psychological (e.g., depression, anxiety), and social factors. Moreover, pain-specific psychosocial constructs, such as catastrophizing, are involved in the development and maintenance of chronic pain and disability (15).

Researchers defined nonorganic signs of pain in 1980, including findings such as non-anatomic tenderness, inconsistent sensory changes, non-replicable pain on distraction tests and pain with simulated movement (16). In a systematic review published in *JAMA,* Chou and Shekelle found that the most useful predictors of developing chronic back pain were “maladaptive pain coping behaviors, nonorganic signs, functional impairment, general health status, and presence of psychiatric comorbidities” (17). The risk factors identified in this study do not include physical signs or findings. Therefore, these findings contradict the theory that chronic pain is primarily caused by a physical lesion, challenging the traditional paradigm. This is further supported by the fact that *nonorganic* signs consistently remain strong predictors of functional performance in patients with low back pain (18) and delay in return to work (19). Furthermore, psychological distress and depression, even in patients without back pain, are associated with the development of future back pain (20, 21), and patients with chronic back pain have substantially higher rates of depression, anxiety, and somatization symptoms compared to patients without chronic pain (22).

Patients with chronic back pain often have additional symptoms and disorders that cannot be explained by the current paradigm of pain. These include headaches, migraines, pain in arms and legs, fibromyalgia, irritable bowel syndrome, complex regional pain syndrome and a number of other medically unexplained syndromes (23–27). While multiple theories have been proposed for the linkage of these syndromes, including centralized changes in pain sensation (28), the reason for the overlap of these conditions is not well understood and cannot be adequately explained by the traditional peripheral injury paradigm. Experimental research on pain supports the theory that the brain can generate pain with little to no peripheral injury or input (29–33).

### 2.3 Current treatment options for chronic pain

#### 2.3.1 Medical treatments

Hundreds of treatments have been suggested for chronic back pain and very few have proven effective. The Cochrane collaboration has concluded that there is little to no high quality evidence to support the use of ultrasound (34), traction (35), spinal manipulation (36), chiropractic treatment (37), transcutaneous electrical nerve stimulation (38), corticosteroid or local anesthetic injections (39), or laser therapy (40) in the assessment and treatment of chronic pain. Moreover, a number of large well-controlled studies, published in the *New England Journal of Medicine,* have not found any benefit of specific exercises or bed rest (41), corticosteroid injections (42), or transcutaneous electrical nerve stimulation (43). Even the use of opioids in pain management (44) has limited long-term efficacy and raises concerns about increased risk to benefit, which are dose-dependent and include worsened mental health, addiction, and death secondary to overdose (45, 46).

Intention-to-treat analysis in trials of surgery for lumbar degenerative spondylolisthesis (47) and sciatica (48) found no difference in the primary outcomes between those randomized to surgical vs. nonsurgical treatment. Additional studies have found no difference in long-term outcomes between spinal fusion and non-operative treatment (49), no clinically meaningful difference (as defined by study investigators) between disc prosthesis and rehabilitation (50) and no difference in outcomes between transpedicular fusion and non-surgical treatment (51). One study reported that back pain outcomes were worse in communities with higher rates of back surgery (52).

#### 2.3.2 Psychological interventions for chronic pain

Learning and cognitive processes such as conditioning and expectation, as well as psychological stress or trauma, appear to play a primary role in predisposing for and maintaining chronic pain. Current research suggests that this connection takes place by creating, activating, and maintaining neural pain pathways (53).

Cognitive behavior therapy (CBT) is the most popular psychotherapy modality for chronic pain. CBT employs a variety of skills including goal setting, activity pacing, environmental changes, attention management, cognitive restructuring, behavioral experiments, and problem solving with the goal to teach clients to successfully self-manage their pain (54). Sveinsdottir et al. (55) found that CBT was beneficial when compared to wait-list controls or treatment as usual but results were mixed and inconclusive when it is compared with other active treatments. In addition to CBT, biofeedback has been found to help in chronic back pain with a meta-analysis finding a small to medium effect size for the reduction of pain intensity (56).

More recent approaches for chronic pain such as mindfulness and acceptance-based interventions emphasize awareness of present experience beyond pain and engaging in values-based activities despite pain (57). Recent evaluations of these approaches suggest that they also surpass treatment as usual but are not more beneficial than CBT (58–60). Nevertheless, mindfulness-based interventions for chronic pain seem to be effective by achieving nonreactivity to cognitions, emotions, and physiological sensations, and can lead to decreased pain catastrophizing and improved quality of life (61, 62). Therefore, practicing mindfulness training may be beneficial by reducing perceived stress and decreasing the severity of chronic pain symptoms (63). Another mechanism by which mindfulness may improve health outcomes is by enhancing the perception of symptom control and a focus on the present (63). A randomized clinical trial investigated the efficacy of Mindfulness-Based Stress Reduction (MBSR) compared to both CBT and usual care in patients with chronic lower back pain, and results showed a comparable reduction in pain between the CBT and MBSR groups, highlighting the potential efficacy of MBSR in the treatment of chronic lower back pain (64).

Overall, existing psychological and behavioral interventions for chronic pain aim to manage pain and help individuals accept, rather than attempt to control, their pain experiences, and appear to have only small to medium effects on pain and disability outcomes. For example, a review by Williams et al., 2020 concluded that CBT has only small to medium effects on pain, disability, and mood when compared to non-active controls, and effects are more limited when CBT is compared with active control conditions (65). Similarly, acceptance and mindfulness-based interventions are most effective compared to non-active controls, and acceptance-based therapies appear to have limited effects on long-term pain severity (66). Overall, though beneficial for some, CBT and mindfulness/acceptance-based interventions alone have limitations in achieving clinically meaningful outcomes for chronic pain patients. One limitation of these treatment approaches is the lack of focus on significant pain reduction or elimination as the primary outcome (with a stronger focus on pain interference). Further, these approaches often do not focus on the potential of the brain to not only modulate pain, but to also generate pain experiences.

In a recent interventional trial similar to our pilot study and our current proposal, researchers found success in psychological treatment for chronic pain by evaluating the efficacy of changing patients' pain-related beliefs–identified by trial investigators as pain reprocessing–compared to both placebo and usual care in patients with chronic back pain (67). The study findings highlighted that nonorganic pain maintained by fear, avoidance of specific activities, and beliefs that pain indicates injury, could successfully be treated through interventions aimed at shifting patients' beliefs about the causes and threat of their pain. Findings from this randomized trial underscore the importance of developing and testing new models and treatment modalities for pain of psychophysiologic origin.

We propose a novel model and treatment approach for chronic back pain of psychophysiologic etiology. Taking into consideration that these patients do not have a significant structural pathology, it may be possible to significantly reduce or eliminate their pain, instead of just attempting to manage the pain (68). The aim of our model and approach is to directly communicate to patients that pain (without clear organic pathology) is generated by the brain, and in turn communicate to patients that significant pain reduction (or even elimination) is possible and achievable.

#### 2.3.3 Proposed psychophysiological mechanism of chronic pain

Our hypothesis can be explained by the following mechanism: psychological stressors create processes in the central nervous system that can lead to changes in peripheral tissue, such as pain. We hypothesize that the underlying etiology of pain is psychophysiological but acknowledge a mechanism by which the body's stress response creates measurable physical changes (such as muscle spasms) could still be present. The effects of psychological processes on physiological changes have been observed in a variety of contexts. For example, embarrassment may result in vasodilation of the capillaries resulting in blushing or sudden traumatic news can result in Takotsubo cardiomyopathy, otherwise known as broken heart syndrome (28). In a pain-related example, Burns et al. have shown that patients with low back pain develop increased activity in the paraspinal muscles when recalling an event that made them angry (69). In other words, back muscles may subconsciously contract in response to anger much as facial muscles contract with various emotions providing an outward recognizable look to others (happiness, anger, sadness). Thus, we hypothesize that this pain syndrome can be treated by addressing the underlying stressors/psychological underpinnings without specific medical interventions. Of note, our hypothesis contrasts with previous descriptions of trigger points where a more bidirectional relationship between peripheral (physical) and central is postulated (11).

## 3 Psychophysiologic symptom relief therapy (PSRT)

Reports on functional or psychogenic backache and backache due to stress factors were reported in the NEJM in 1946 after observing soldiers returning from the battlefield during World War I (9). The notion that chronic pain may originate from organic causes but persists as a manifestation of displaced/repressed feelings (e.g., hostility or aggressive impulses that the individual is unwilling or unable to acknowledge) was formulated in 1959 (14). More recent research has shown that the individual experience of perceived pain is shaped by past pain exposures, stress responses, cognitions, and emotions (53) as well as established the link between pain and psychopathology (70).

The reports of Sarno (71) as well as the Explaining Pain model by Moseley and Butler (72) suggest that, at least for types of pain with no clear organic etiology, participants may benefit from an explanatory model that stresses that their pain is of psychophysiological origin. Treatment should focus on learning experiences allowing participants to become more aware of emotional processes–potentially leading to pain remission or elimination. Thus, we developed a treatment program for such participants with non-specific chronic back pain. PSRT is a novel pain reduction intervention that builds on a psychophysiologic (mind-body) approach to understanding and treating chronic pain. The intervention is based on Dr. John Sarno's hypothesis that chronic pain lacking an identified organic etiology is a physical manifestation of primarily psychological processes (12). He hypothesizes chronic pain can be treated without targeted physical interventions by addressing underlying stressors and psychological conflicts. Findings from previous small research studies and case series support the hypothesis that treatment methods targeting psychological processes can lead to improvement in functioning and pain levels for chronic pain patients without obvious organic causes (12, 68, 73, 74). A related approach by Lumley et al. focusing on emotional expression (Emotion Awareness and Expression Therapy; EAET), resulted in better outcomes for overall symptoms–widespread pain, physical functioning and negative affect–compared to the education control group in a randomized trial with fibromyalgia patients (75). The results from additional randomized trials evaluating EAET efficacy for patients with medically unexplained symptoms and pain conditions including irritable bowel syndrome and chronic urogenital pain indicate that it is effective in reducing pain and other somatic symptoms and improving physical functioning (76–79).

An important pillar of PSRT is to address and to challenge fear-avoidance behaviors and beliefs. According to the classical conditioning model, previously neutral stimuli can be linked to the pain experience. Thus, without the need for a current nociceptive input, this association can be coupled with physical reactions ultimately leading to pain. For example, formerly elicited pain-induced muscle tensions are associated with other stimuli like bending over, or walking. Even the thought of these activities could result in anxiety and therefore increased muscle tension. This fear of movement, or *kinesiophobia,* and the associated fear of pain, are important factors in the development of chronic pain (80, 81).

### 3.1 Components of the treatment program

We have developed a program, PSRT, consisting of four major components: increasing knowledge of the link between the mind and pain (psychophysiologic pain education), desensitization (including visualization techniques), encouraging emotional awareness/expression (decreasing emotional avoidance or repression), and improving stress reduction skills (i.e., mindfulness meditation).

The goal of PSRT is to address underlying stressors and psychological contributors (such as underlying conflicts and aversive affective states) to nonspecific symptoms in order to mitigate conditioned symptom responses and fear-avoidant behaviors. The first 4 weeks of the treatment include group classes twice per week (90–120 min per class) and consist of psychophysiologic education, desensitization (including visualization techniques), and emotional awareness exercises. Education focuses on providing patients with information about the role of stress and psychological processes in precipitating and perpetuating physical symptoms. Using desensitization exercises, participants are encouraged to approach, rather than avoid, physical activities first through visualization (e.g., visualizing an action that typically induces symptoms) and then through physical exposures of feared symptom-inducing activities (e.g., bending). The emotional awareness (e.g., expressive writing) exercises are introduced during the educational component of PSRT, and are utilized throughout the treatment. The final 9 weeks of PSRT consist of a Mindfulness Based Stress Reduction (MBSR) protocol as outlined by the Center for Mindfulness at the University of Massachusetts (82). This portion consists of classes once per week for a duration of 90 to 120 min and focuses on providing participants with mindfulness skills such as practicing awareness of breath, body scan, and sitting meditation. Further details of the PSRT protocol can be found in the published pilot trial manuscript (83).

Of note, PSRT shares similarities with Pain Reprocessing Therapy (PRT), a program tested in a recently published clinical trial discussed above (67). While PRT is similar to PSRT with some components, there are some differences. For example, PRT consisted of one-on-one psychotherapy with a therapist for four weeks whereas PSRT was a structed interactive didactic program in small group settings. The focus of PRT was on “retraining” the brain through reframing a patients' orientation toward pain with an emphasis on techniques such as somatic tracking. The study protocol stated that addressing underlying emotional components was an adjunctive technique. In contrast, while psychoeducational and reframing are part of PSRT, the program also included a strong emphasis on emotional expression techniques, achieved mostly through a series of different journaling exercises. Both programs include re-engagement of activities.

### 3.2 PSRT pilot study results

Our team conducted a pilot study to assess whether PSRT can reduce disability and back pain bothersomeness for patients with chronic back pain (83). The pilot study was a three-armed, randomized trial for adults with non-specific chronic back pain comparing PSRT to MBSR and usual care. As noted in the original published report (83), participants were excluded if the study team identified organic sources of pain such as but not limited to malignancy, neurologic disorder (e.g., amyotrophic lateral sclerosis and cauda equine syndrome), or findings of severe spinal stenosis. Patients with disc disease were not necessarily excluded (unless there were neurological impairments) but were taken into consideration with their overall history and depending on the location/nature of the pain. Participants randomized to PSRT received a 12 week (36 h) course based on the psychophysiologic model of pain. Participants randomized to the active comparator arm underwent an 8-week group-based MBSR program. Validated surveys were administered to all groups at baseline and at 4, 8, 13, and 26 weeks. The primary outcome was the reduction in pain disability measured by the Roland Disability Questionnaire (RDQ). The mean RDQ for the PSRT group (*n* = 11) decreased from 9.5 (±4.3) to 3.3 (±5.1) after 26 weeks which was statistically significant compared to both MBSR (*n* = 12) (*p* = 0.04) and usual care (*n* = 12) (*p* = 0.03). In the MBSR group the RDQ decreased from 12.6 (±5.8) to 8.2 (±6.7) and in the usual care group it remained nearly unchanged 8.7 (±6.4) to 8.5 (±7.6) after 26 weeks. Pain bothersomeness scores and pain-related anxiety decreased significantly over 26 weeks in the PSRT group compared to the MBSR and usual care groups. The results of the pilot study suggest that PSRT is a feasible and potentially efficacious treatment for patients with non-specific chronic back pain (83).

### 3.3 PSRT clinical trial

We are currently executing a prospective randomized interventional trial (*N* = 150) with three study arms to evaluate PSRT as a treatment for chronic back pain lacking an organic etiology (NCT04689646). The trial design is similar to the pilot study in that participants in the treatment arm will receive PSRT, while those in the baseline control arm will proceed with their usual care. The third study arm will serve as the “active comparator” in which participants will receive mindfulness-based stress reduction, providing an internal standard for the intervention. Pain disability (primary outcome), back pain bothersomeness, and pain anxiety will be assessed to further evaluate the efficacy of PSRT for chronic back pain.

## 4 Conclusion

In sum, we hypothesize that non-specific chronic back pain may result from an underlying psychophysiologic process such that psychological stressors result in the physiological output of pain. PSRT is a program designed to treat the root of the problem and therefore will test this hypothesis. The full development of this hypothesis as discussed in this manuscript and the promising results of our pilot study provide the rationale for an ongoing large randomized trial evaluating PSRT.
